# Comparative Performance of AI Models and Clinicians in Evidence-Based Cardiovascular Disease Management for People Living With HIV: Comparative Study

**DOI:** 10.2196/89858

**Published:** 2026-08-10

**Authors:** Tianqi Kong, Liqin Sun, Yinsong Luo, Xi Xiao, Jin Li, Jiaye Liu

**Affiliations:** 1 School of Public Health, Shenzhen University Medical School, Shenzhen University, No.1066 Xueyuan Avenue, Shenzhen, Guangdong, 518060, China, 86 18819026906; 2 Department of Infectious Diseases, National Clinical Research Center for Infectious Diseases, Shenzhen Third People’s Hospital, Shenzhen, Guangdong, China; 3 Department of Infectious Diseases, The Ninth People's Hospital of Dongguan, Dongguan, Guangdong, China

**Keywords:** HIV, cardiovascular disease, AI, comorbidity management, patient education

## Abstract

**Background:**

Although widespread antiretroviral therapy has extended the life expectancy of people living with HIV, cardiovascular disease (CVD) has emerged as a primary comorbidity. Persistent cross-specialty knowledge gaps in routine clinical practice lead to suboptimal adherence to guidelines. Integrated, evidence-based tools are urgently needed to overcome these interdisciplinary barriers. While large language models (LLMs) have demonstrated significant capabilities in medicine, no systematic evaluation has assessed their ability to facilitate multidisciplinary CVD management for people living with HIV.

**Objective:**

This study compared the performance of 4 mainstream AI models (DeepSeek-V3, DeepSeek-R1, ChatGPT-4o, and ChatGPT-o4-mini) against 12 human clinicians (8 infectious disease specialists and 4 cardiologists) in addressing guideline-based CVD management tasks for people living with HIV.

**Methods:**

Based on 4 authoritative domestic and international HIV/CVD guidelines, a structured 25-question assessment was developed via 2 rounds of Delphi consultation. Standard reference answers and an evaluation framework were finalized through expert consensus. LLM responses were generated using standardized prompts. Clinicians answered identical questions via one-on-one structured interviews, transcribed verbatim. Six multidisciplinary experts independently rated all responses across 4 dimensions—accuracy, completeness, readability, and reliability—using a 4-point ordinal scale (1=poor to 4=excellent). Cumulative link mixed models analyzed intergroup differences.

**Results:**

All AI models achieved significantly higher scores than clinicians across all dimensions (*P*<.001). The AI group’s mean scores ranged from 3.44 to 3.68 (median 4, IQR 3.0-4.0; coefficient of variation=0.145-0.178). Conversely, clinicians’ scores were lower (mean 1.78-2.05; median 2, IQR 1.0-3.0; coefficient of variation=0.428-0.473) with marked dispersion. DeepSeek-R1 delivered the optimal performance, significantly outperforming the other 3 models (all *P*<.001). Specialty-stratified analysis revealed no significant overall score difference between cardiologists and infectious disease specialists (odds ratio 0.92, 95% CI 0.84-1.01; *P*=.09). However, dimension-specific analysis indicated that cardiologists scored higher in accuracy (odds ratio 0.81, 95% CI 0.67-0.97; *P*=.03). Domain-specific divergence was evident: cardiologists outperformed infectious disease specialists in CVD risk assessment (2.26 vs 1.83), whereas infectious disease specialists led in drug adverse effect evaluation (2.23 vs 1.65).

**Conclusions:**

In this structured question-and-answer study, LLMs outperformed human clinicians across all metrics for HIV-associated CVD management, with DeepSeek-R1 achieving superior composite scores. These findings validate DeepSeek-R1’s potential as a cross-disciplinary decision-support tool capable of integrating complex clinical knowledge, mitigating specialty gaps, and enhancing information precision. Integrating AI systems into multidisciplinary workflows, complemented by targeted clinical training, may optimize the management of complex comorbidities in people living with HIV.

## Introduction

HIV infection remains a major global public health challenge. By the end of 2024, approximately 40.8 million people were living with HIV worldwide [1]. The widespread use of antiretroviral therapy (ART) has significantly improved life expectancy among people living with HIV [2]. However, non-AIDS–defining conditions, particularly cardiovascular disease (CVD), are increasingly recognized as critical factors affecting both quality of life and long-term prognosis [3-5]. Epidemiological studies indicate that the risk of CVD among people living with HIV is approximately twice that of the general population, with onset occurring nearly a decade earlier on average [6,7]. The recent REPRIEVE (Randomized Trial to Prevent Vascular Events in HIV) randomized trial further shifted the landscape by demonstrating that proactive statin therapy reduces major adverse cardiovascular events in people living with HIV regardless of traditional risk score thresholds [8], highlighting the need for structured prevention approaches in this population.

In this context, effective CVD management has become a key priority in improving health outcomes among people living with HIV. However, significant interdisciplinary knowledge gaps persist in clinical practice: infectious disease clinicians often lack cardiovascular expertise, while cardiologists may be unfamiliar with ART-related drug interactions. These limitations hinder shared decision-making in areas such as medication selection, risk stratification, and long-term follow-up, potentially leading to suboptimal treatment decisions and adverse clinical outcomes. Traditional medical training has not kept pace with the complexity of HIV-related comorbidities, highlighting the urgent need for innovative tools to support consistent, evidence-based, multidisciplinary care.

Among these innovative tools, large language models (LLMs) have recently shown great promise in health care applications [9-11]. Built on the transformer architecture, LLMs can process complex language patterns and learn medical knowledge from large-scale textual data [12]. Several studies have shown that AI models can generate clinically accurate and interpretable content across diverse specialties [13-15]. Nonetheless, their utility in managing complex, overlapping comorbidities—such as HIV and CVD—remains poorly understood. Most prior studies have focused on single disease scenarios [16-18], and few have examined whether LLMs can synthesize interdisciplinary knowledge, bridge cognitive gaps across specialties, and provide reliable decision support. Notably, different models vary in clinical reasoning depth and update efficiency [19-21], which may impact their real-world applicability.

This study aims to systematically compare the performance of 4 mainstream LLMs—DeepSeek-V3, DeepSeek-R1, ChatGPT-4o, and ChatGPT-o4-mini—with that of human clinicians in addressing guideline-based CVD management tasks for people living with HIV. We evaluated 4 question categories (basic knowledge, drug management, clinical decision-making, and case analysis) and assessed each response along 4 dimensions: accuracy, completeness, readability, and reliability. By doing so, this study seeks to determine whether LLMs can effectively bridge interdisciplinary knowledge gaps and serve as reliable decision-support tools for managing complex comorbidities in people living with HIV.

## Methods

### Overview

**Figure 1.**
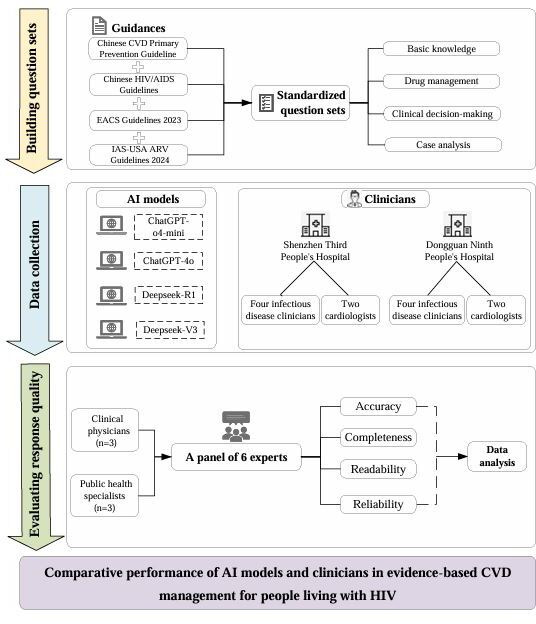
Flowchart of overall study design. CVD: cardiovascular disease; EACS: European AIDS Clinical Society.

This cross-sectional comparative study systematically evaluated the performance of 4 AI models (DeepSeek-V3, DeepSeek-R1, ChatGPT-4o, and ChatGPT-o4-mini) and 12 clinicians in responding to CVD management questions among people living with HIV. A standardized question set was developed based on authoritative clinical guidelines to ensure consistency across all evaluations (Figure 1).

### Question Set Development

To ensure both the authority and timeliness of the question set, we selected 4 evidence-based guidelines as references. These included the Guidelines for Primary Prevention of Cardiovascular Diseases in China [22] and the Chinese Guideline for Diagnosis and Treatment of HIV/AIDS (2024 Edition) [23], which reflect local clinical practice, as well as 2 internationally recognized guidelines: the 2023 European AIDS Clinical Society Guidelines [24] and Antiretroviral Drugs for Treatment and Prevention of HIV in Adults: 2024 Recommendations of the International Antiviral Society-USA Panel [25]. All 4 were developed by leading academic institutions in the fields of CVD and HIV/AIDS, incorporating the most recent high-quality clinical evidence.

Based on the core content of these guidelines, we adopted a dual-framework strategy that integrates knowledge dimension stratification with clinical needs orientation to screen and classify the questions. Concurrently, we used a 2-round Delphi expert consultation method to systematically refine and optimize the question set. In the first round, 10 experts from multiple disciplines, including cardiology, infectious diseases, and public health, were invited to rate 35 candidate questions across 4 dimensions—importance, feasibility, answer clarity, and expression clarity—using a 1‐5 Likert scale, with open-ended suggestions also solicited. Based on the feedback from the first round, we merged, added, and deleted questions to form a standardized set of 25 questions. In the second round of Delphi consultation, the same panel of experts was asked to rerate each question in the revised set (using the same dimensions) and simultaneously evaluate the predefined reference answers in terms of reasonableness and guideline conformity (also using a 1‐5 Likert scale). Ultimately, questions with mean scores ≥4.0 and coefficients of variation (CVs) ≤0.25 across all dimensions were retained, and textual optimization suggestions from the experts were adopted, resulting in a final structured question set of 25 items.

The questionnaires used in the 2 rounds of Delphi surveys are detailed in Multimedia Appendix 1.

This standardized question set follows a progressive framework from basic theory to practical application and then to comprehensive clinical decision-making, covering all key elements of CVD management in people living with HIV. The specific categories are as follows (Table 1): the basic theory category focuses on theoretical understanding, assessing respondents’ mastery of fundamental concepts and standards of CVD management in HIV care; the medication management category addresses 2 common clinical challenges: interactions between ART and cardiovascular drugs, as well as adverse metabolic effects associated with ART; the clinical decision-making category concentrates on formulating diagnostic and treatment plans under specific clinical scenarios, requiring the integration of multidimensional knowledge; and the case analysis category evaluates respondents’ ability to conduct comprehensive clinical reasoning through complex real-world cases, including risk assessment, treatment planning, and management strategy optimization.

After the second round of Delphi expert consultation, we finalized reference answers to ensure objective and consistent evaluation. These answers were formulated by prioritizing authoritative guidelines, following a clear hierarchy: Chinese guidelines were prioritized over international guidelines, and guidelines specific to people living with HIV were prioritized over those for the general population, based on the study population’s characteristics. We then considered the levels of evidence and clarified points of controversy. This standardized reference then served as the benchmark for evaluation.

**Table 1.** Structured question set on CVD^a^ management in people living with HIV based on authoritative guidelines^b^.

Category and subcategory	Questions
Basic knowledge	
CVD risk assessment	1. What cardiovascular risk assessment tools are currently available, and are they applicable to people living with HIV? 2. How is cardiovascular risk stratified into low, medium, and high categories? 3. What are the common cardiovascular risk factors specific to people living with HIV?
Lifestyle behaviors	4. What is the recommended upper limit of daily salt intake to reduce CVD risk? 5. What proportion of carbohydrates should be consumed by people living with HIV with elevated blood sugar risk? 6. What level of physical activity is recommended for people living with HIV to reduce CVD risk? 7. How does sleep affect cardiovascular risk, and what are the criteria for healthy sleep?
Health indicator control	8. What are the target LDL-C^c^ levels for people living with HIV with intermediate, high, and very high cardiovascular risk? 9. What are the recommended blood pressure targets for people living with HIV classified as high cardiovascular risk? 10. What are the optimal blood glucose control standards for people living with HIV to prevent CVD? 11. How frequently should blood pressure and blood glucose be monitored in people living with HIV?
Drug management	
Drug interactions	12. Which ART^d^ regimens may interact with statins? 13. What are the key considerations when coadministering rilpivirine with other cardiovascular medications, such as calcium channel blockers? 14. What are the risks of drug interactions between tenofovir and diuretics? 15. How do protease inhibitors in ART regimens influence the effectiveness of oral hypoglycemic agents, such as sulfonylureas and metformin?
Side effects of drugs	16. Which ART regimens should be avoided in patients with HIV at high risk of CVD? 17. Which ART options may contribute to increased LDL-C levels? 18. Which ART regimens are most likely to cause weight gain?
Clinical decision-making	
—^e^	19. What are the preferred hypoglycemic treatments for people living with HIV who are at high cardiovascular risk and have type 2 diabetes? 20. What are the stepwise management strategies for people living with HIV with varying blood pressure levels? 21. What are the indications for initiating lipid-lowering therapy in people living with HIV? 22. What lipid-lowering strategies are recommended for patients with different levels of renal dysfunction? 23. What are the causes and management strategies for central adiposity in postmenopausal women receiving dolutegravir-based ART?
Case analysis	
Case 1	24. A 45-year-old male with a 10-year history of HIV infection, on ART (EFV^f^+TDF^g^+FTC^h^-based regimen). Current immune status: CD4^i^+580 cells/μL, HIV RNA persistently<20 copies/mL (for 3 years). Persistent dyslipidemia (uncontrolled for 3 years): TC^j^ 5.2 mmol/L, LDL-C 3.4 mmol/L, HDL-C^k^ 1.0 mmol/L, TG^l^ 1.8 mmol/L; clinic BP^m^ 130/85 mm Hg, home-measured average BP 128/82 mm Hg; FPG^n^ 5.8 mmol/L, HbA_1c_^o^ 5.6%; BMI 26 kg/m^2^, waist circumference 94 cm, eGFR^p^ 92 mL/minute/1.73 m^2^. Framingham 10-year risk score 12%, CAC^q^ score 50. History of smoking (10 years, 20 pack-years), quit 2 years ago; sedentary office job, fast food–based diet. What are the contributors to dyslipidemia in this patient? Is ART implicated? Does this patient meet indications for initiating lipid-lowering therapy? Design a lipid-lowering regimen including drug selection, dosing, and monitoring plan. Propose nonpharmacologic strategies to improve cardiovascular risk in this patient.
Case 2	25. A 58-year-old woman with a 15-year history of HIV infection. Initial ART regimen: LPV/r^r^ + TDF/FTC (continued for 13 years). Switched to DTG^s^ + 3TC 2 years ago due to mixed hyperlipidemia (peak values: TC 7.2 mmol/L, LDL-C 5.0 mmol/L, TG 4.8 mmol/L). Concurrent conditions: Type 2 diabetes (5-year duration, HbA_1c_ 7.5%), currently on metformin 1000 mg bid; hypertension (8-year duration, BP 140-150/85-95 mm Hg), on amlodipine. Latest fasting lipids: TC 6.0 mmol/L, LDL-C 4.0 mmol/L, HDL-C 1.1 mmol/L, TG 2.5 mmol/L; renal function: eGFR 68 mL/minute/1.73 m^2^, UACR^t^ 32 mg/g. Cardiovascular risk scores: Framingham 28%, ASCVD^u^ 10-year risk 20%, CAC score 450. BMI 28 kg/m^2^, sedentary lifestyle, waist circumference 92 cm. Based on CVD risk stratification tools (eg, Framingham, ASCVD, and CAC), how should this patient’s risk be categorized? What is the target LDL-C for this patient? If statin monotherapy fails to achieve target, what are appropriate combination therapy strategies? Assess the justification for the simplified DTG+3TC regimen considering viral suppression, metabolic effects, and drug interactions.

a CVD: cardiovascular disease.

b This table, based on authoritative guidelines, systematically compiles a structured set of questions regarding the management of CVD in people living with HIV.

c LDL-C: low-density lipoprotein cholesterol.

d ART: antiretroviral therapy.

e Not available.

f EFV: efavirenz.

g TDF: tenofovir disoproxil fumarate.

h FTC: emtricitabine.

i CD4+: cluster of differentiation 4 (a subtype of T-lymphocytes).

j TC: total cholesterol.

k HDL-C: high-density lipoprotein cholesterol.

l TG: triglyceride.

m BP: blood pressure.

n FPG: fasting plasma glucose.

o HbA_1c_: hemoglobin A_1c_.

p eGFR: estimated glomerular filtration rate.

q CAC: coronary artery calcium.

r LPV/r: lopinavir/ritonavir.

s DTG: dolutegravir.

t UACR: urinary albumin-to-creatinine ratio.

u ASCVD: atherosclerotic cardiovascular disease.

### AI Model Selection and Response Acquisition

We selected 4 representative LLMs, namely, DeepSeek-V3, DeepSeek-R1, ChatGPT-4o, and ChatGPT-o4-mini. DeepSeek-V3 is a universal basic model suitable for handling daily tasks; DeepSeek-R1 specializes in complex reasoning and in-depth analytical tasks; ChatGPT-4o features multimodal capabilities and excels in handling routine workflows; and ChatGPT-4o-mini demonstrates notable strengths in science, technology, engineering, and mathematics-related tasks. All models were accessed independently via their respective official public interfaces or clients.

To ensure consistent and standardized assessment, a unified prompting framework was established for the AI model. The core stipulations embedded in the system prompt included assigning the model the identity of an HIV-specialized clinician, defining 4 categories of research questions (basic knowledge, medication management, clinical decision-making, and case analysis), requiring the model to learn 4 designated clinical guidelines, and setting a fixed evidence hierarchy to resolve inconsistent recommendations across guidelines. The full text of the standardized system prompt is provided in Multimedia Appendix 2 for reference.

Subsequently, the 25 structured questions were input verbatim into each model without any additional instructions attached to individual queries, ensuring that all models received identical information. Each question was submitted only once to avoid potential bias from prior interactions or model memory. Finally, the complete text responses for all 25 questions were collected from each model for subsequent evaluation.

### Clinician Recruitment and Response Acquisition

We recruited 12 clinicians from 2 designated hospitals for HIV care in Guangdong Province, China: Shenzhen Third People’s Hospital and Dongguan Ninth People’s Hospital. The inclusion criteria were as follows: (1) holding a valid medical license and having at least 3 years of clinical experience, (2) being familiar with the diagnosis and treatment of either HIV infection or CVD, and (3) providing informed consent for voluntary participation. Based on their clinical specialties, the clinicians were categorized into 2 groups, with 8 in the infectious diseases group and 4 in the cardiology group. There were no statistically significant differences between the 2 groups in terms of professional title or years of clinical experience, ensuring comparability.

To balance the response conditions between the AI model and clinicians and control confounding bias, all interviews were conducted in quiet office settings as structured face-to-face verbal question-and-answer sessions with audio recording equipment, which also accommodated clinicians’ work schedules and facilitated natural responses from participants. Prior to each interview, researchers read a standardized introductory script to participating physicians uniformly (see Multimedia Appendix 3 for details). The script covered the study purpose, response restrictions (no access to any paper or electronic external resources throughout the interview), 4 reference guidelines with a predefined hierarchy of evidence, and classification criteria for the 4 major question categories. Formal questioning only commenced after physicians fully acknowledged comprehension of all rules and granted consent for audio recording. Each interview was scheduled to last 30-60 minutes, while the actual response duration ranged from 20 to 45 minutes (median 30 minutes).

All audio recordings were transcribed verbatim without subjective alterations to preserve original content. Transcripts were cross-checked by 2 independent researchers to guarantee accuracy and completeness. Any ambiguous or vague statements were verified against the original audio recordings to confirm the intended meaning. To mitigate information bias, transcription and coding were performed under a double-blind protocol; all researchers were blinded to participants’ identity information and group assignments throughout the entire process.

### Evaluation Criteria and Implementation

We constructed a multidimensional evaluation framework encompassing 4 core dimensions: accuracy, completeness, readability, and reliability. The determination of these dimensions was jointly established by multiple experts with clinical and research experience on the research team, through extensive literature searches and reviews followed by several rounds of collective discussion. Specifically, accuracy measures the degree of correctness of medical facts in the response; completeness assesses whether the response covers all key elements involved in the question; readability concerns whether the language expression is clear and the structure is reasonable; and reliability examines whether the response provides reliable guideline-based evidence or a sound clinical reasoning process. These 4 dimensions complement one another and aim to comprehensively characterize the quality of responses from different perspectives. Each dimension was rated on a 4-point scale, where 4 represented the highest performance and 1 the lowest. This system was designed to provide a thorough and detailed evaluation of the responses generated for each question (Table 2).

For the 2 complex case-based questions, which require the integration of multidimensional knowledge and comprehensive clinical reasoning, a single-dimensional score cannot adequately reflect their inherent hierarchical structure. Therefore, based on expert discussion, we developed independent weighted scoring schemes for these 2 case questions (Table 3). In these schemes, each subquestion was assigned a different weight according to its clinical relevance and contribution to the overall decision, ensuring that higher-priority elements contributed more substantially to the total score. The specific weight values were also collectively determined by the expert panel through reference to relevant literature and clinical practice consensus. To enhance the clarity and discriminability of the scoring process, we established a standardized rule for handling scores falling between 2 integers—when a score lay between adjacent integers, the midpoint of the interval was used as the cutoff value to guide the final scoring decision. This approach facilitated more robust between-group statistical comparisons in subsequent analyses.

Following the establishment of the evaluation criteria, we convened a panel of 6 experts, comprising 3 public health specialists and 3 clinical physicians. All panel members were independent of the question design and data collection phases. Prior to scoring, the evaluators underwent standardized training to ensure a consistent understanding and application of the scoring criteria. The assessment was conducted using a blinded, independent review protocol. All response texts were anonymized in advance by removing identifying information such as AI model names, clinician names, and institutional affiliations, and each entry was assigned a unique code. Experts then independently evaluated the responses without knowledge of their origin. Upon completion of individual assessments, all score sheets were retrieved and reviewed for interrater consistency.

**Table 2.** Definition of evaluation dimensions.

Assessment dimensions and standard description	Score setting
Accuracy	
The degree to which the answer content aligns with guideline-based evidence, without scientific errors.	4=Fully accurate, with no medical errors, and strictly adheres to guideline recommendations. 3=Generally accurate, with only minor expression issues that do not affect the main conclusion. 2=Factually correct overall, but contains evident errors or misleading details. 1=Marginally relevant or contains fundamental scientific inaccuracies.
Completeness	
The extent to which the response covers all key points of the question.	4=Comprehensive and well-supported response covering all relevant aspects of the question. 3=Covers the main topic, but lacks elaboration or omits some secondary details. 2=Oversimplified answer with missing explanation or ≥2 key elements omitted. 1=Substantial omissions; addresses only a small portion of the question.
Readability	
The clarity, coherence, and ease of understanding of the response, facilitating clinical application.	4=Well-structured, with appropriate use of bullet points or tables, accurate terminology, and no redundant information. 3=Clear and fluent, though may contain minor segmentation issues or a small amount of irrelevant detail. 2=Logically inconsistent or difficult to follow; requires rereading to grasp. 1=Vague or confusing expression, hindering comprehension.
Reliability	
The degree to which the response relies on authoritative sources and aligns with established guidelines.	4=Explicitly references high-priority, authoritative guidelines and clearly reflects their content. 3=Consistent with guideline principles but lacks clear attribution to specific sources. 2=Partially aligns with recommendations; relies on less authoritative or unspecified sources. 1=Conflicts with guideline content or lacks credible basis.

**Table 3.** Weighted scoring framework for case analysis questions based on clinical importance.

Case analysis question	Core component	Weight (%)	Weighted score (score×weight)
Question 24			
What are the contributors to dyslipidemia in this patient? Is ART^a^ implicated?	Etiological analysis	20	0.8
Does this patient meet indications for initiating lipid-lowering therapy?	Treatment indications	20	0.8
Design a lipid-lowering regimen including drug selection, dosing, and monitoring plan.	Therapeutic options	40	1.6
Propose nonpharmacologic strategies to improve cardiovascular risk in this patient.	Nonpharmaceutical intervention	20	0.8
Question 25			
Based on CVD^b^ risk stratification tools (eg, Framingham, ASCVD^c^, and CAC^d^), how should this patient’s risk be categorized?	Risk stratification	20	0.8
What is the target LDL-C^e^ for this patient?	Key health indicators	20	0.8
If statin monotherapy fails to achieve target, what are appropriate combination therapy strategies?	Combination pharmacotherapy	30	1.2
Assess the justification for the simplified DTG^f^+3TC^g^ regimen considering viral suppression, metabolic effects, and drug interactions.	Overall clinical decision plan	30	1.2

a ART: antiretroviral therapy.

b CVD: cardiovascular disease.

c ASCVD: atherosclerotic cardiovascular disease.

d CAC: coronary artery calcium.

e LDL-C: low-density lipoprotein cholesterol.

f DTG: dolutegravir.

g TC: total cholesterol.

### Statistical Analysis

All data analyses were performed using R software (version 4.4.2; R Foundation for Statistical Computing). The primary outcome was the expert-assigned rating scores, which were treated as ordinal categorical variables. To assess data distribution, the Shapiro-Wilk test was used to examine normality, and the Levene test was applied to assess the homogeneity of variances. Descriptive statistics including median and IQR, as well as mean and SD, were used to summarize overall and group-specific performance. Furthermore, to account for the hierarchical structure of the data while respecting the ordinal nature of the outcome variable, we constructed a cumulative link mixed model (CLMM) using the *ordinal* package in R. The model included group membership (AI models vs clinicians) as a fixed effect and random intercepts for both question and rater to capture the clustering of responses. The proportional odds assumption was verified via a likelihood-ratio test comparing models with and without nonproportional odds terms. Post-hoc pairwise comparisons among groups (eg, different AI models) were conducted using the Tukey method for CLMM contrasts, with adjustment for multiple testing.

To assess the reliability of the expert ratings, interrater agreement among the 6 evaluators was evaluated using the intraclass correlation coefficient (ICC), based on a 2-way random-effects model. Specifically, ICC(2,1) was used to assess the reliability of individual raters, while ICC(2,k) reflected the reliability of the average scores across the panel. An ICC value greater than 0.75 was interpreted as indicating good consistency.

All statistical tests were 2-sided, and a *P* value of less than .05 was considered statistically significant.

### Ethical Considerations

This study received ethics approval from the medical ethics committee of the Medical School of Shenzhen University (approval: PN-202500127). Given that this study solely used anonymous structured interviews with clinicians and a comparative evaluation of responses generated by AI models, no patient samples, clinical medical records, or sensitive personally identifiable information were collected throughout the study. The medical ethics committee approved the waiver of written informed consent. All research procedures were performed in strict accordance with the ethical principles outlined in the Declaration of Helsinki.

## Results

### Performance Comparison Between AI Models and Clinicians

This study evaluated the performance of 4 AI models (ChatGPT-4o, ChatGPT-o4-mini, DeepSeek-V3, and DeepSeek-R1) against 12 clinicians (comprising cardiologists and infectious disease clinicians) in answering 25 structured questions related to CVD management in people living with HIV. Each response was independently assessed by 6 experts across 4 core dimensions: accuracy, completeness, readability, and reliability.

**Figure 2.**
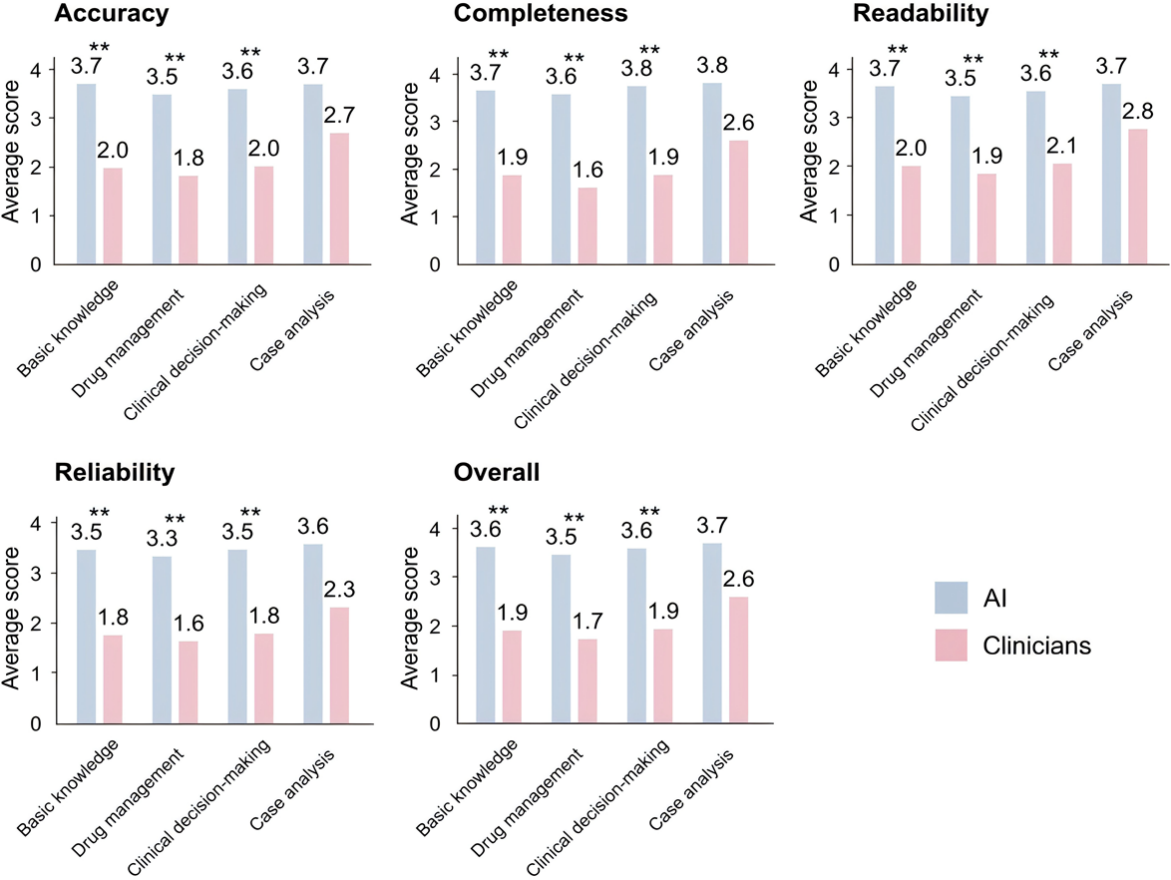
Comparison of performance between the AI model and clinicians across different question categories and evaluation dimensions. The AI model (blue bars) demonstrated significantly higher overall scores compared to clinicians (pink bars) in the majority of categories and dimensions. Error bars represent SD. Asterisks indicate a significant overall effect of the rater group (AI vs clinician) based on a cumulative link mixed model (**P<.001).

Descriptive analysis showed that the AI group had mean scores ranging from 3.44 to 3.68 across the 4 dimensions, with a median of 4.0 (IQR 3.0-4.0) and CV between 0.145 and 0.178, reflecting relatively high performance consistency. In contrast, the clinician group had mean scores between 1.78 and 2.05, a median of 2.0 (IQR 1.0-2.0), and CV values from 0.428 to 0.473. Across all 4 question types—basic knowledge, drug management, clinical decision-making, and case analysis—the AI group obtained higher scores than the clinician group. The highest scoring dimension for the AI group was completeness in case analysis questions (mean score 3.8, SD 0.39), while the lowest for clinicians was in drug management (mean score 1.6, SD 0.83). Detailed results are presented in Table 4 and Figure 2.

To formally evaluate differences while accounting for the hierarchical structure of the data (responses nested within questions and raters) and the ordinal nature of the Likert scale, a CLMM was fitted using restricted maximum likelihood. The model demonstrated good fit, with random intercepts for “Question” (variance=0.0795) and “Expert” (variance=0.0723), indicating moderate clustering effects. In the fixed effects analysis, the intercept was estimated at −4.422 (SE 0.117; *P*<.001). The coefficient for the clinician group was −5.066 (SE 0.121; *P*<.001), corresponding to a significantly lower likelihood of achieving higher scores. Specifically, the odds of the AI group receiving a higher score category compared to the clinician group were 83.6 times greater (odds ratio [OR] 0.012, 95% CI 0.010‐0.014; *P*<.001).

**Table 4.** Comparison of overall distribution between AI models and clinicians.

Group and dimension	Mean (SD)	Range	Median (IQR)	CV^a^
AI				
Accuracy	3.63 (0.554)	1-4	4 (3-4)	0.152
Completeness	3.68 (0.532)	1-4	4 (3-4)	0.145
Readability	3.59 (0.586)	1-4	4 (3-4)	0.163
Reliability	3.44 (0.612)	1-4	4 (3-4)	0.178
Clinicians				
Accuracy	2.00 (0.855)	1-4	2 (1-3)	0.428
Completeness	1.87 (0.884)	1-4	2 (1-2)	0.473
Readability	2.05 (0.929)	1-4	2 (1-3)	0.454
Reliability	1.78 (0.774)	1-4	2 (1-2)	0.435

a CV: coefficient of variation.

### Performance Comparison Among Different AI Models

**Figure 3.**
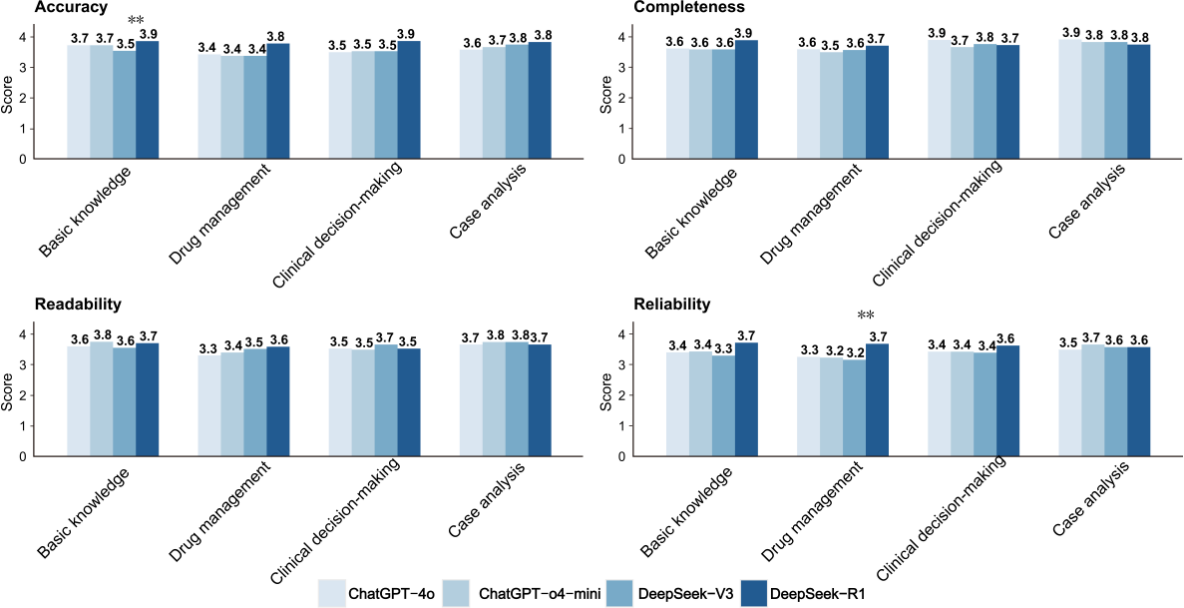
Comparative performance evaluation of 4 AI models. Error bars represent SD, with 4 question types contributing to the mean for each dimension. Significance markers indicate pairwise comparisons based on Tukey-adjusted contrasts from the cumulative link mixed model. **P<.001*.*

The performance of 4 AI models was further compared across 4 evaluation dimensions and 4 question categories. Descriptive statistics showed that DeepSeek-R1 achieved the highest scores across most evaluation dimensions, particularly in accuracy, with mean scores between 3.79 and 3.87. It performed particularly well in the basic knowledge questions (accuracy: 3.86; completeness: 3.89). Among the comparison group of the other 3 models, the overall performance was largely comparable. ChatGPT-o4-mini exhibited a nuanced advantage in handling complex questions, slightly outperforming others in accuracy (3.73) and readability (3.76) for basic knowledge questions, as well as in completeness (3.83) and readability (3.75) for case analysis questions. This aligns with its ability in managing complex scenarios (Figure 3).

To formally evaluate differences among the 4 AI models while accounting for the hierarchical data structure and the ordinal nature of the outcome, a CLMM was fitted, followed by post-hoc pairwise comparisons using the Tukey method. The CLMM revealed a significant overall effect of model type (likelihood-ratio test: *c*^2^=24.346; *P*<.001). Post-hoc comparisons showed a clear tiered differentiation in performance. DeepSeek-R1 demonstrated a substantial advantage over both ChatGPT-o4-mini and DeepSeek-V3. Specifically, DeepSeek-R1 had significantly higher odds of receiving a higher score compared to ChatGPT-o4-mini (OR 2.47, 95% CI 1.90‐3.21; *P*<.001) and 2.53 times higher than those of DeepSeek-V3 (estimate=0.929; OR 2.53, 95% CI 1.94‐3.31; *P*<.001). In contrast, pairwise comparisons among ChatGPT-4o, ChatGPT-o4-mini, and DeepSeek-V3 revealed no statistically significant differences across any of the evaluated dimensions (adjusted *P* values ranged from .55 to 0.99). For example, the comparison between ChatGPT-4o and ChatGPT-o4-mini yielded an estimate of −0.008 (OR 0.992, 95% CI 0.78-1.27; *P*=.99). These results indicate that DeepSeek-R1 occupies a distinct top tier, while the other 3 models perform at a comparable level.

### Comparison of Performance Between Different Clinician Groups

**Figure 4.**
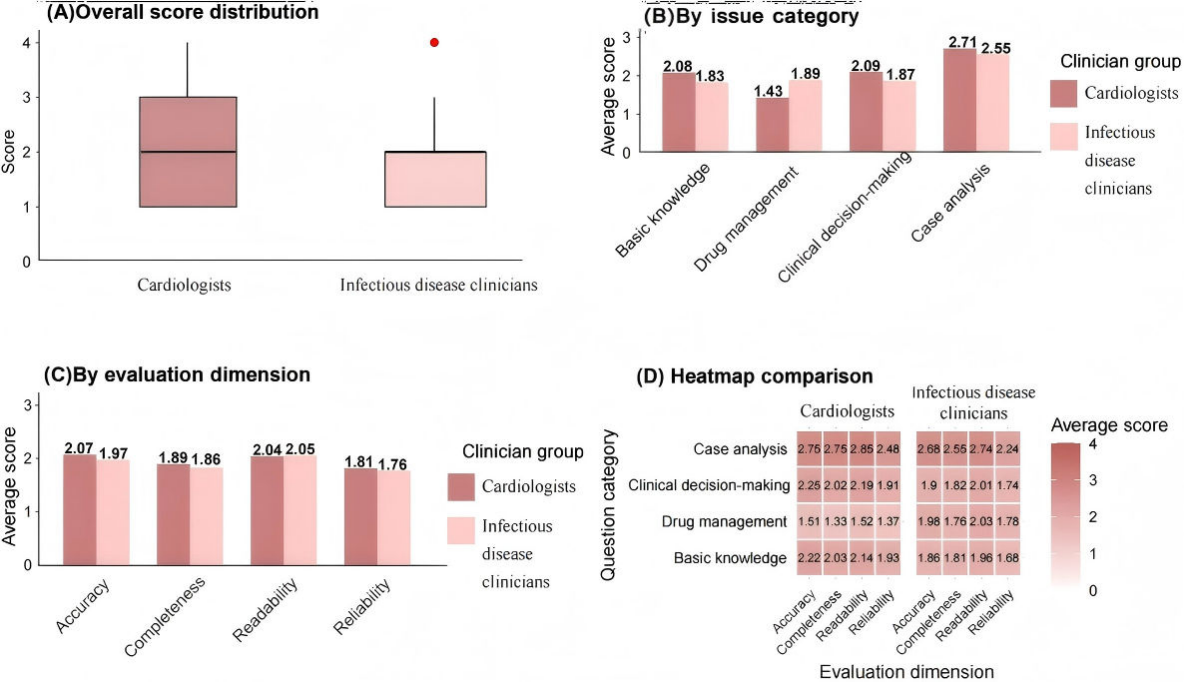
Multifaceted comparison of responses among different groups of clinicians. (A) Overall score distribution presented as a boxplot. Mean scores stratified by (B) question category and (C) evaluation dimension. (D) Heat map of scores across categories and dimensions. Group comparisons were performed using a cumulative link mixed model with Tukey-adjusted post-hoc tests (see the Methods section for details).

We also compared the performance of cardiologists (n=4) and infectious disease clinicians (n=8) across overall scores, question categories, and evaluation dimensions. Descriptive analysis (consistent with Shapiro-Wilk test results indicating nonnormal distributions: cardiologists *W*=0.837; *P*<.001; infectious disease clinicians *W*=0.830; *P*<.001) showed comparable central tendencies: the median overall score was 2 (IQR 1.0-3.0) for cardiologists and 2 (IQR 1.0-2.0) for infectious disease clinicians (Figure 4A). To formally evaluate group differences while accounting for the ordinal nature of the outcome and hierarchical data structure, a CLMM was fitted, followed by Tukey post-hoc pairwise comparisons. The CLMM revealed a significant main effect of clinician group (likelihood-ratio test: *c*^2^ (1)=19.34; *P*<.001).

**Figure 5.**
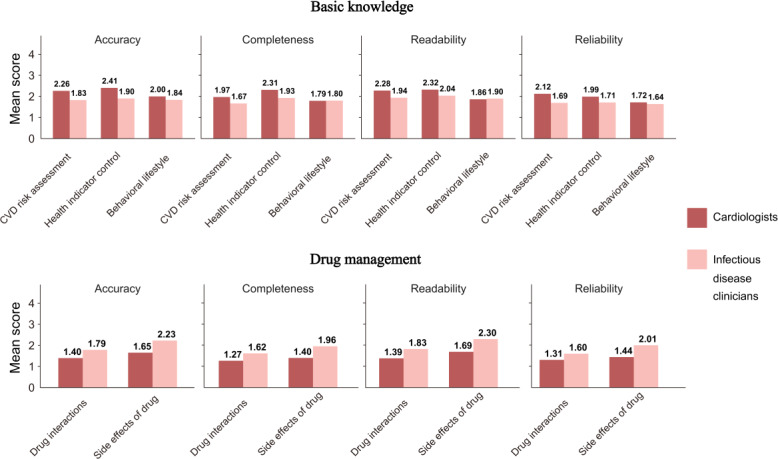
Performance comparison of different clinician groups across various problem categories. Data are presented as mean scores for each problem category. Group comparisons were conducted using a cumulative link mixed model with Tukey-adjusted post-hoc tests (see the Methods section for details). CVD: cardiovascular disease.

Post-hoc comparisons highlighted domain-specific strengths (Figure 4B-D). In basic knowledge questions, cardiologists scored significantly higher than infectious disease clinicians (median difference 0.25; OR 2.41, 95% CI 1.83‐3.17; *P*<.001), driven by superior performance in CVD risk assessment (accuracy: median 2.26, IQR 1.0-3.0 vs median 1.83, IQR 1.0-3.0; Figure 5). Conversely, infectious disease clinicians excelled in drug management (median difference −0.46; OR 0.41, 95% CI 0.31‐0.54; *P*<.001), particularly in managing ART side effects (accuracy: median 2.23, IQR 1.0-3.0 vs median 1.65, IQR 1.0-2.0; Figure 5). In clinical decision-making, there was no statistically significant difference between the groups (median difference 0.22; OR 2.09, 95% CI 1.60‐2.74; adjusted *P*=.16). Similarly, in case analysis, the difference (median difference 0.16; OR 1.71, 95% CI 1.03‐2.85) also remained nonsignificant after multiple comparison correction (*P*_adj_≥.99 for accuracy).

By evaluation dimension (Figure 4C and D), no significant group differences emerged (clinical decision-making: adjusted *P*=.16; case analysis: adjusted *P*≥.99). For accuracy, cardiologists had marginally higher scores (median 2.07, IQR 1.0-3.0 vs median 1.96, IQR 1.0-2.0), but this was not statistically significant (*P*_adj_=.09). Similarly, completeness (median difference=0.02; *P*_adj_=.67), readability (median difference=−0.09; *P*_adj_=.52), and reliability (median difference=0; *P*_adj_=.14) showed no meaningful divergence. A heat map (Figure 4D) further illustrated these specialty-specific patterns: cardiologists consistently scored higher on cardiovascular-related items (eg, CVD risk assessment and health indicator control), whereas infectious disease clinicians excelled in pharmacotherapy-focused tasks (eg, drug interactions and side effect management).

### Interrater Consistency Analysis

Interrater agreement was quantified using ICC (Table 5). For single-rater reliability (ICC(2,1)), both accuracy (ICC=0.755, 95% CI 0.712-0.794) and completeness (ICC=0.754, 95% CI 0.711-0.793) exceeded the commonly accepted threshold of 0.75 (*P*<.001), indicating acceptable consistency among individual expert ratings. In contrast, readability (ICC=0.681) and reliability (ICC=0.713) demonstrated only moderate agreement (*P*<.001), suggesting greater variability in individual assessments of these 2 dimensions.

**Table 5.** Analysis of interrater consistency (n=6 experts).

Dimensions	ICC^a^(2,1) (95% CI)	ICC(2,k) (95% CI)	*P* value
Accuracy	0.755 (0.712-0.794)	0.949 (0.937-0.960)	<.001
Completeness	0.754 (0.711-0.793)	0.949 (0.937-0.960)	<.001
Readability	0.681 (0.629-0.729)	0.927 (0.910-0.942)	<.001
Reliability	0.713 (0.664-0.758)	0.937 (0.923-0.949)	<.001

a ICC: intraclass correlation coefficient.

To assess the robustness of aggregated expert scoring, average-measures ICC (ICC(2,k)) was further calculated. When ratings were averaged across all 6 experts, excellent consistency was observed across all dimensions: accuracy (ICC=0.949, 95% CI 0.937-0.960), completeness (ICC=0.949, 95% CI 0.937-0.960), readability (ICC=0.927, 95% CI 0.910-0.942), and reliability (ICC=0.937, 95% CI 0.923-0.949; all *P*<.001). All values were well above the 0.90 threshold, confirming high reliability of group-based scoring outcomes.

## Discussion

### Principal Findings

This study presents one of the first comprehensive, head-to-head comparisons between LLMs and human clinicians in the context of managing CVD in people living with HIV. By evaluating performance across multiple clinical domains and rating dimensions, our findings consistently demonstrate the superior capability of LLMs in generating accurate, complete, and clinically coherent responses, even within the complex interdisciplinary setting of HIV-CVD comorbidity. This performance advantage was observed across all question types, from basic knowledge to real-world case analysis, and was particularly evident in areas requiring integrative reasoning. Importantly, this advantage emerged despite the AI models receiving only a minimal system prompt specifying their role and the relevant guidelines, rather than being explicitly instructed to mimic a clinician. This suggests that the models’ pretraining on vast medical corpora inherently equips them with the knowledge and reasoning patterns necessary for complex comorbidity management, underscoring their readiness as decision-support tools.

This superiority stems from 2 fundamental advantages of LLMs: robust logical reasoning and broad knowledge coverage [26-28]. Built on advanced algorithmic architectures such as transformers, these models are capable of mining and extracting key features from massive repositories of medical literature, clinical guidelines, consensus statements, and case-based evidence [29,30]. This data-driven integration enables LLMs to conduct multisource, multidimensional information synthesis within milliseconds, allowing them to maintain consistency, accuracy, and completeness in complex clinical scenarios [31]—qualities that are often compromised in human decision-making due to cognitive biases, fatigue, and fragmented training. These results suggest that, when appropriately deployed, LLMs may serve as a powerful tool for bridging cognitive gaps between specialties and standardizing care quality in settings with limited access to multidisciplinary expertise.

### Clinical Implications

It is important to address the clinical relevance of the observed statistical differences. On our 4-point ordinal scale, a score of 4 represents “fully accurate, with no medical errors, and strictly adheres to guideline recommendations,” while a score of 2 indicates “factually correct overall, but contains evident errors or misleading details.” The mean AI scores of 3.44‐3.68 therefore correspond to responses that are near-perfect in guideline adherence, whereas clinician mean scores of 1.78‐2.05 indicate responses that, on average, contain noticeable inaccuracies or omissions. This qualitative gap has direct implications for patient safety and care quality: even small numerical differences on this scale can translate into clinically meaningful distinctions, such as the difference between recommending a contraindicated drug combination versus a guideline-concordant alternative.

Furthermore, the extremely low CV in the AI group (CV 0.145‐0.178) compared with clinicians (CV 0.428‐0.473) underscores a critical advantage of AI: its ability to deliver uniformly high-quality advice regardless of question complexity or domain. In real-world settings, this consistency can reduce the “knowing-doing gap” and mitigate variability in clinical decision-making—a known driver of disparate patient outcomes. The clinical relevance is most pronounced in the context of complex comorbidities like HIV and CVD, where interdisciplinary knowledge silos are common. An AI model that integrates cardiology and infectious disease guidelines can directly support a clinician in making a safer, more holistic decision than they might achieve alone, particularly in resource-limited settings without easy access to multidisciplinary teams.

These findings also hint at an underlying complementarity between clinical specialties that merits closer examination, as discussed in the following section.

### Performance Heterogeneity

Notable performance differences among the 4 AI models highlight the impact of training strategies and model architecture on clinical reasoning outcomes. DeepSeek-R1 consistently outperformed other models, particularly in tasks requiring factual precision and logical integration, such as basic knowledge and drug safety. This may reflect its specialized training focus on reasoning-intensive tasks and optimized instruction-tuning processes, which likely enhanced its ability to synthesize structured clinical information [32]. It is important to note that all models were tested under identical conditions (same prompt and default temperature settings), so the observed differences can be attributed primarily to architectural and training differences rather than prompt engineering.

In contrast, general-purpose models like ChatGPT-4o and DeepSeek-V3 demonstrated relatively balanced but less domain-specific performance, while ChatGPT-o4-mini showed modest advantages in case analysis, possibly due to its science, technology, engineering, and mathematics–oriented pretraining [33]. These variations suggest that the clinical utility of LLMs is closely tied to the relevance and quality of their underlying training data. Domain-adaptive pretraining and instruction tuning—especially using medical guidelines, real-world case repositories, and multiturn clinical dialogue—may significantly improve model performance in complex decision-making scenarios.

From a translational perspective, these findings underscore the importance of aligning model selection with task characteristics in real-world deployment. For settings requiring high interpretability and decision fidelity, domain-optimized models like DeepSeek-R1 may be preferable. Future efforts should focus on dynamic fine-tuning, multilingual optimization, and integration with local clinical knowledge systems to enhance the safety, generalizability, and cultural adaptability of AI-assisted tools across health care environments.

### Cross-Specialty Complementarity

Despite the overall performance gap between AI models and human clinicians, our analysis of subgroup differences revealed meaningful patterns within the clinician group itself [34]. Notably, while cardiologists and infectious disease clinicians achieved comparable total scores, their strengths diverged across task types. Cardiologists performed better in cardiovascular risk assessment and complex clinical decision-making, whereas infectious disease clinicians outperformed in drug management tasks, particularly in addressing side effects of drugs. These differences likely reflect each group’s clinical focus and training background—cardiologists are more experienced with risk stratification algorithms and long-term prognosis planning, while infectious disease clinicians are more attuned to the nuances of ART regimens, metabolic complications, and drug-drug interactions.

Such findings underscore the existence of “knowledge silos.” These silos are precisely the gaps that LLMs, with their ability to retrieve and synthesize cross-disciplinary knowledge, can help bridge. This divergence also reinforces the need for more integrated, team-based approaches to care. In real-world practice, the absence of consistent cross-specialty collaboration may lead to fragmented decision-making and missed opportunities for optimized management. From an educational and clinical workflow perspective, integrating LLMs into multidisciplinary team discussions could provide real-time, guideline-aligned suggestions that supplement each specialists’ blind spots, potentially reducing fragmentation and improving care coordination [35].

Moreover, the observed complementarity between specialties hints at a potential role for LLMs as integrative tools in bridging these knowledge gaps [36,37]. AI-driven decision support systems could offer real-time, cross-domain insights that align with both infectious disease and cardiovascular best practices, thereby supporting more cohesive clinical strategies [38]. Future research should explore how such tools can be embedded within multidisciplinary workflows to promote collaborative decision-making and improve outcomes in complex care scenarios.

### Methodological Contributions

Beyond the performance findings, this study offers methodological value for future evaluations of AI-assisted clinical decision support. Unlike previous studies that relied on simplified question-answer prompts or single-dimension metrics [39], we adopted a standardized, guideline-derived question set, incorporated structured case scenarios, and implemented a multidimensional evaluation framework to capture both the breadth and depth of clinical reasoning. The use of expert-developed reference answers ensured consistency in benchmarking, and the blinded scoring protocol minimized observer bias. Furthermore, we involved experts from both public health and clinical specialties. The interrater agreement, quantified through ICC analysis, demonstrated excellent agreement for average scores across all dimensions (ICC=0.93‐0.95). These measures ensured that the evaluation process was both reliable and generalizable. This design may serve as a practical assessment paradigm for future studies seeking to objectively measure the clinical utility of LLMs in complex, multidisciplinary contexts.

### Limitations

Despite its strengths, this study has several limitations. First, the sample size for clinician participants was relatively small, with only 12 clinicians from 2 HIV-designated hospitals in the same region (Guangdong Province, China). Although we ensured comparable experience levels between specialties and adopted a blinded evaluation approach, the findings may not be generalizable to broader clinical populations or health care systems, and the geographic concentration limits external validity. Second, each AI model was prompted only once per question under standardized conditions, which may not fully reflect their potential performance variability or capacity for interactive refinement—especially in real-world deployments where multiturn dialogues are common. Furthermore, the system prompt used (assigning the model the role of a clinician and specifying relevant guidelines) represents just one possible prompting strategy; alternative prompts could lead to different performance outcomes. Third, while our question set was carefully constructed based on authoritative guidelines, it cannot capture the full complexity and uncertainty of clinical practice, such as patient heterogeneity, comorbidities beyond HIV and CVD, or emerging clinical scenarios. Fourth, the weighting scheme for case analysis questions, although determined by expert consensus, introduces inherent subjectivity. Different weighting priorities (eg, emphasizing diagnostic accuracy over treatment planning) could alter the relative rankings of groups. Sensitivity analyses using alternative weighting methods would strengthen the robustness of our findings. Fifth, the evaluation framework, although multidimensional and rigorously implemented, remains inherently subjective, particularly in dimensions such as readability or reliability. Despite acceptable interrater reliability (single-rater ICC=0.68‐0.76; average-rater ICC=0.93‐0.95), some degree of rater bias is unavoidable. Finally, the assessment focused on textual response quality and did not evaluate downstream clinical outcomes, patient safety, or acceptability of AI integration in practice.

### Conclusions

This study provides the first systematic comparison of LLMs and human clinicians in addressing CVD management for people living with HIV. The results show that AI models achieved significantly higher scores across all evaluation dimensions while also revealing complementary strengths between specialties. These findings highlight the potential of LLMs as decision-support tools that can augment, rather than replace, clinical expertise—particularly in complex comorbidity contexts where cross-specialty knowledge integration is essential. The strong domain-specific performance of DeepSeek-R1 further suggests that model selection should consider contextual alignment with real-world clinical needs, beyond size or architecture alone. Future work should prioritize prospective clinical validation and the integration of AI into multidisciplinary workflows, combining human judgment with machine precision to ensure safe and effective deployment.

## Supplementary material

10.2196/89858 Multimedia Appendix 1 Construction of a structured question set for CVD management in people living with HIV: A two-round Delphi expert consultation.

10.2196/89858 Multimedia Appendix 2 Full AI system prompt.

10.2196/89858 Multimedia Appendix 3 Standardized opening script for clinician face-to-face interviews.

## Abbreviations

ART: antiretroviral therapy
CLMM: cumulative link mixed model
CV: coefficient of variation
CVD: cardiovascular disease
ICC: intraclass correlation coefficient
LLM: large language model
OR: odds ratio
REPRIEVE: Randomized Trial to Prevent Vascular Events in HIV
