# Supplementary material for: Comparative Performance of AI Models and Clinicians in Evidence-Based Cardiovascular Disease Management for People Living With HIV: Comparative Study
Source: J Med Internet Res. 2026 Aug 10;28:e89858. doi: 10.2196/89858 (PMC13456308; doi:10.2196/89858)
Supplement: Supplementary file 1 [file jmir-v28-e89858-s001.pdf]

Construction of a Structured Question Set for CVD Management in People Living with HIV
First-Round Delphi Expert Consultation

Dear Expert/Professor:

We cordially invite you to participate in this Delphi expert consultation on "Construction of a Structured Question Set for CVD Management in People Living with HIV".

With the widespread adoption of antiretroviral therapy (ART), the life expectancy of people living with HIV (PLWH) has approached that of the general population. However, non-AIDS-defining conditions such as cardiovascular disease (CVD) have become a major challenge affecting their long-term health and quality of life. Managing HIV-CVD comorbidity requires deep integration of multidisciplinary knowledge from infectious disease and cardiology, yet cross-disciplinary knowledge barriers are commonly encountered in clinical practice. To explore the potential of artificial intelligence (AI) as a decision-support tool in this complex comorbidity management scenario, we have initiated a study aimed at systematically evaluating the performance of AI models and clinicians in responding to HIV-CVD management questions.

The foundation and core of this study is the construction of an authoritative, structured, and clinically relevant question set to serve as a standardized tool for evaluating the performance of AI models and clinicians. To this end, based on the latest domestic and international HIV and CVD prevention and treatment guidelines (including the Chinese Guidelines for Primary Prevention of Cardiovascular Disease, Chinese Guidelines for Diagnosis and Treatment of AIDS, the European AIDS Clinical Society (EACS) Guidelines, and the U.S. Department of Health and Human Services (DHHS) Guidelines for the Use of Antiretroviral Agents in Adults and Adolescents with HIV), we have preliminarily drafted a key question list covering five dimensions: "CVD Risk Assessment," "Lifestyle Intervention," "Indicator Control," "Pharmacotherapy," and "Mental Health."

To ensure the scientific validity and clinical utility of this question set, and its ability to effectively differentiate the knowledge depth and decision-making capabilities of different respondents (AI versus clinicians, and clinicians from different specialties), we are conducting this round of Delphi expert consultation. Your professional judgment is essential for screening core questions, optimizing question phrasing, and ensuring consistency with guidelines and relevance to clinical practice. The question set formed after your review will be directly used for the subsequent standardized evaluation

of AI models and clinicians, and its quality is directly related to the reliability and generalizability of our study conclusions.

This consultation is expected to consist of two rounds. The current questionnaire aims to collect your assessments of the scientific validity, feasibility, answer definitiveness, and clarity of expression of each question. We sincerely request that you kindly review and complete this questionnaire amidst your busy schedule; your valuable input is crucial to the rigorous conduct of this study.

We assure you that all information obtained from this consultation will be used solely for this study, and your personal information will be kept strictly confidential.

We sincerely thank you for your support and contribution!

I. Basic Information of the Expert

1. Your name: _________________________;

   Age: ________ ;

   Gender: □ Male □ Female

2. Your institution: ____

3. Your position/title: ____

4. Your highest educational degree:

   □ Doctoral degree □ Master's degree □ Bachelor's degree □ Other

5. Your specialty and field of expertise (multiple choices allowed):

   □ Infectious Disease/HIV Specialty □ Cardiology/Cardiovascular Prevention □ Clinical Pharmacy □ Endocrinology/Metabolic Diseases □ Nursing/Health Education □ Other (please specify): _________________

6. Years of professional experience: _________ years

7. Have you published HIV- or CVD-related papers or participated in the development of relevant guidelines or consensus statements in the past 5 years? □ Yes □ No

II. Instructions for Completion

Please rate the importance, feasibility, answer definitiveness, and clarity of expression for each question (quantitative values are shown in Table 1), and indicate your level of familiarity

(quantitative values are shown in Table 1). Please enter the corresponding quantitative values in the table.

If you believe a question should be deleted or revised, please state this in the "Revision Suggestions" column of the table. If you believe additional questions should be added, please enter them in the * column and rate their importance, feasibility, answer definitiveness, clarity of expression, and familiarity.

Table S1 Quantitative Scales for Indicator Evaluation and Level of Familiarity

Importance	Score	Feasibility	Score	Answer Definiteness	Score	Clarity of Expression	Score
Very important	5	Very feasible	5	Very definite	5	Very clear	5
Important	4	Feasible	4	Definite	4	Clear	4
Moderately important	3	Moderately feasible	3	Moderately definite	3	Moderately clear	3
Less important	2	Less feasible	2	Less definite	2	Less clear	2
Not important	1	Not feasible	1	Not definite	1	Not clear	1

Note: Scores are discrete values. Answer definitiveness refers to whether the question has a relatively clear evidence-based or expert consensus answer, facilitating subsequent evaluation of the accuracy of AI and clinician responses. Clarity of expression refers to whether the question is stated clearly and unambiguously, directly affecting the operability of subsequent evaluations.

Example:

Question	Importance (1-5)	Feasibility (1-5)	Answer Definiteness (1-5)	Clarity of Expression (1-5)	Revision Suggestions
A11 What are the specific methods for CVD risk assessment in PLWH?	5	5	5	4	It is recommended to add a description of the applicable population for "lifetime risk assessment".

Example explanation: The rater assigned scores of 5, 5, 5, and 4 for the importance, feasibility, answer definiteness, and clarity of expression of indicator A11, respectively. The rating was primarily based on "practical experience + theoretical analysis." The rater was familiar with indicator A11, with a corresponding score of 0.8. For indicator A11, it is suggested to supplement the description of the applicable population for lifetime risk assessment in the question phrasing.

III. First-Round Delphi Expert Consultation Form

Table S2 First-Level Indicator Evaluation Form

First-Level Indicator	Importance (1-5)	Feasibility (1-5)	Answer Definiteness (1-5)	Clarity of Expression (1-5)	Revision Suggestions
A CVD Risk Assessment (Questions 1-9)					
B Lifestyle Intervention (Questions 10-19)					
C Indicator Control (Questions 20-24)					
D Pharmacotherapy (Questions 25-32)					
E Mental Health (Questions 33-35)					

Dimension Descriptions:

A CVD Risk Assessment: Methods, tools, risk stratification, and screening strategies for assessing cardiovascular disease risk in PLWH. Includes overall risk assessment procedures, commonly used assessment tools (FRS, SCORE, ASCVD, D:A:D, etc.), risk factor identification, and screening frequency.

B Lifestyle Intervention: Reducing CVD risk in PLWH through dietary adjustment, physical activity, weight management, smoking cessation and alcohol limitation, and sleep improvement. Includes dietary recommendations, exercise prescriptions, and BMI control targets.

C Indicator Control: Monitoring targets and control standards for CVD-related biochemical and clinical indicators in PLWH. Includes target values for blood lipids (LDL-C, TC, TG, HDL-C), blood glucose (fasting blood glucose, glycated hemoglobin), and blood pressure, as well as risk stratification.

D Pharmacotherapy: ART regimen selection and optimization, lipid-lowering drug use, drug-drug interactions, and adherence management. Includes ART drugs associated with weight gain/dyslipidemia, recommended ART regimens, statin selection and dose adjustment, and strategies for missed doses.

E Mental Health: Assessment and intervention for the psychological burden faced by PLWH regarding CVD comorbidity risk. Includes coping with dual psychological burden, correct understanding of abnormal indicators, the impact of psychological status on CVD risk, and mitigation strategies.

Table S3 Second-Level Indicator Evaluation Form

First-Level Indicator	Second-Level Indicator	Importance (1-5)	Feasibility (1-5)	Answer Definiteness (1-5)	Clarity of Expression (1-5)	Revision Suggestions
A CVD Risk Assessment	A1 What are the specific methods for CVD risk assessment in PLWH?					
	A2 What are the commonly used cardiovascular disease assessment tools? Are they applicable to the HIV population?					
	A3 Why is the D:A:D model recommended for assessing cardiovascular disease risk in the HIV population?					
	A4 Which category of PLWH is at higher cardiovascular disease risk?					
	A5 Are PLWH more likely to develop cardiovascular disease than the general population? Why?					
	A6 Should PLWH undergo cardiovascular disease-related screening annually?					
	A7 Which traditional cardiovascular disease risk factors are more common among PLWH?					
	A8 What is the impact of antiretroviral therapy on cardiovascular disease risk?					
	A9 What are the risk factors for cardiovascular disease in the HIV population?					

First-Level Indicator	Second-Level Indicator	Importance (1-5)	Feasibility (1-5)	Answer Definiteness (1-5)	Clarity of Expression (1-5)	Revision Suggestions
B Lifestyle Intervention	B10 Which types of food should PLWH increase their intake of to reduce cardiovascular disease risk?					
	B11 Which types of food should PLWH reduce or avoid to lower ASCVD risk?					
	B12 What is the maximum recommended daily salt intake to reduce cardiovascular disease risk?					
	B13 According to the "Chinese Dietary Guidelines for Residents," what are the recommended daily intakes of grains and tubers, vegetables, and fruits for PLWH?					
	B14 What level of physical activity should adult PLWH achieve to reduce the risk of cardiovascular disease?					
	B15 What forms of physical activity are recommended for elderly PLWH?					
	B16 Weight gain and obesity are relatively common after ART initiation in PLWH. What BMI values indicate overweight or obesity? How much weight loss is recommended?					
	B17 Does secondhand smoke exposure increase cardiovascular risk? How long after smoking cessation can cardiovascular disease risk be reduced in PLWH who smoke?					
	B18 Can controlling alcohol intake reduce the risk of cardiovascular events in PLWH? What is the maximum daily alcohol intake?					
	B19 Does sleep affect cardiovascular disease risk? What constitutes healthy sleep?					
C Indicator Control	C20 Which indicators are the primary measures for assessing CVD risk in the HIV population?					
	C21 Some ART drugs may cause					

First-Level Indicator	Second-Level Indicator	Importance (1-5)	Feasibility (1-5)	Answer Definiteness (1-5)	Clarity of Expression (1-5)	Revision Suggestions
	dyslipidemia in PLWH; which specific indicators are affected?					
	C22 What are the cholesterol-lowering targets for patients with different levels of ASCVD risk?					
	C23 What are the indications for initiating lipid-lowering therapy in PLWH?					
	C24 What should the low-density lipoprotein cholesterol (LDL-C) level be controlled below in PLWH? What are the risk stratification criteria?					
D Pharmacotherapy	D25 Which drugs in ART treatment are prone to cause weight gain in PLWH?					
	D26 What ART regimen is recommended for PLWH at higher cardiovascular disease risk?					
	D27 Which antiretroviral drugs in ART regimens may cause side effects such as dyslipidemia and metabolic disorders?					
	D28 For a patient with dyslipidemia whose original ART regimen is based on RTV, COBI booster, or EFV, what conversion recommendations would you propose to optimize the ART regimen?					
	D29 What are the commonly used lipid-lowering drug classes in the HIV population?					
	D30 Can ART regimen drugs and statins produce adverse reactions? Which specific drug combinations have unfavorable interactions?					
	D31 If a PLWH is taking lopinavir/ritonavir, what precautions should be taken when using statins?					
	D32 What are the consequences if a PLWH forgets to take medication? How should this be managed?					

First-Level Indicator	Second-Level Indicator	Importance (1-5)	Feasibility (1-5)	Answer Definiteness (1-5)	Clarity of Expression (1-5)	Revision Suggestions
E Mental Health	E33 PLWH are at higher risk of cardiovascular disease than the general population. How should they cope with and alleviate the dual psychological burden of HIV infection and chronic disease?<br>E34 PLWH may face dyslipidemia due to ART treatment. How should they correctly understand the situation when indicators are not well controlled?<br>E35 Can a good psychological state reduce cardiovascular disease risk? What is the impact?					

End of Form — Thank You for Your Support!

Construction of a Structured Question Set for CVD Management in People Living with HIV
Second-Round Delphi Expert Consultation

Dear Expert/Professor:

First and foremost, we would like to once again express our sincere gratitude for your invaluable comments and suggestions during the first-round consultation. This is the second round of the Delphi consultation, in which we invite you to evaluate the optimized question items and their corresponding reference answers. The evaluation dimensions include: importance of the question, feasibility, answer definiteness, clarity of expression, as well as the reasonableness and guideline concordance of the reference answers. All evaluations adopt a 1–5 point Likert scale, and you are welcome to provide additional comments regarding textual revisions or content adjustments.

The information collected in this consultation will be used solely for academic research purposes, and your personal information will be kept strictly confidential. We sincerely thank you for your support and assistance! This consultation is expected to consist of two rounds. The current questionnaire aims to collect your assessments of the scientific validity, feasibility, answer definiteness, and clarity of expression of each question. We sincerely request that you kindly review and complete this questionnaire amidst your busy schedule; your valuable input is crucial to the rigorous conduct of this study.

We assure you that all information obtained from this consultation will be used solely for this study, and your personal information will be kept strictly confidential.

We sincerely thank you for your support and contribution!

I. Basic Information of the Expert

1. Your name: __________________________;

   Age: _________ years;

   Gender: □ Male □ Female

2. Your institution: ____

3. Your position/title: ____

4. Your highest educational degree:

□ Doctoral degree □ Master's degree □ Bachelor's degree □ Other

5. Your specialty and field of expertise (multiple choices allowed):

□ Infectious Disease/HIV Specialty □ Cardiology/Cardiovascular Prevention □ Clinical Pharmacy □ Endocrinology/Metabolic Diseases □ Nursing/Health Education □ Other (please specify): _________________

6. Years of professional experience: __________ years

7. Have you published HIV- or CVD-related papers or participated in the development of relevant guidelines or consensus statements in the past 5 years? □ Yes □ No

II. Instructions for Completion

Please rate each item sequentially on the following six indicators: importance of the question itself, feasibility, answer definiteness, clarity of expression, as well as the reasonableness and guideline concordance of the accompanying reference answer. Scores range from 1 to 5 points; please enter the corresponding numbers in the table. If you identify issues with the question phrasing, content design, or reference answer, please provide detailed explanations in the "Revision Suggestions" column; additional content may be noted as supplements. Scoring criteria are detailed in the table below, with discrete quantitative values.

Table S1 Quantitative Scales for Indicator Evaluation

Importance	Score	Feasibility	Score	Answer Definiteness	Score	Clarity of Expression	Score	Reference Answer Reasonableness	Score	Guideline Concordance	Score
Very important	5	Very feasible	5	Very definite	5	Very clear	5	Very reasonable	5	Highly concordant	5
Important	4	Feasible	4	Definite	4	Clear	4	Reasonable	4	Concordant	4
Moderately important	3	Moderately feasible	3	Moderately definite	3	Moderately clear	3	Moderately reasonable	3	Moderately concordant	3
Less important	2	Less feasible	2	Less definite	2	Less clear	2	Less reasonable	2	Less concordant	2
Not important	1	Not feasible	1	Not definite	1	Not clear	1	Not reasonable	1	Not concordant	1

Note: Scores are discrete values. Answer definiteness refers to whether the question has a relatively clear evidence-based or expert consensus answer, facilitating subsequent evaluation of the accuracy of AI and clinician responses. Clarity of expression refers to whether the question is stated clearly and unambiguously, directly affecting the operability of subsequent evaluations. Reference answer reasonableness refers to whether the answer is logically rigorous and contentually sound. Reference answer guideline concordance refers to whether it aligns with authoritative guideline requirements.

Example:

Question	Importance (1-5)	Feasibility (1-5)	Answer Definiteness (1-5)	Clarity of Expression (1-5)	Reference Answer Reasonableness (1-5)	Guideline Concordance (1-5)	Revision Suggestions
A11 What are the specific methods for CVD risk assessment in PLWH?	5	5	5	4	5	5	It is recommended to add a description of the applicable population for "lifetime risk assessment".

Example explanation: The rater assigned scores of 5, 5, 5, and 4 for the importance, feasibility, answer definiteness, and clarity of expression of indicator A11, respectively. The rating was primarily based on "practical experience + theoretical analysis." The rater was familiar with indicator A11, with a corresponding score of 0.8. For indicator A11, it is suggested to supplement the description of the applicable population for lifetime risk assessment in the question phrasing.

III. Second-Round Delphi Expert Consultation Form

Table S2 First-Level Indicator Evaluation Form

First-Level Indicator	Importance (1-5)	Feasibility (1-5)	Answer Definiteness (1-5)	Clarity of Expression (1-5)	Revision Suggestions
A Basic Knowledge (CVD Risk Assessment, Behavioral Lifestyle, Health Indicator Control)					
B Drug Management (Drug-Drug Interactions, Drug Side Effects)					
C Clinical Decision-Making					
D Case Analysis					

Table S3 Second-Level Indicator Evaluation Form

First-Level Indicator	Second-Level Indicator	Importance (1-5)	Feasibility (1-5)	Answer Definiteness (1-5)	Clarity of Expression (1-5)	Revision Suggestions
A Basic Knowledge	1. What cardiovascular risk assessment tools are currently available, and are they applicable to PLWH?					
	2. How is cardiovascular risk stratified into low, medium, and high categories?					
	3. What are the common cardiovascular risk factors specific to PLWH?					

First-Level Indicator	Second-Level Indicator	Importance (1-5)	Feasibility (1-5)	Answer Definiteness (1-5)	Clarity of Expression (1-5)	Revision Suggestions
	4. What is the recommended upper limit of daily salt intake to reduce CVD risk?					
	5. What proportion of carbohydrates should be consumed by HIV-positive individuals with elevated blood glucose risk?					
	6. What level of physical activity is recommended for PLWH to reduce CVD risk?					
	7. How does sleep affect cardiovascular risk, and what are the criteria for healthy sleep?					
	8. What are the target LDL-C levels for HIV-infected patients with intermediate, high, and very high cardiovascular risk?					
	9. What are the recommended blood pressure targets for PLWH classified as high cardiovascular risk?					
	10. What are the optimal blood glucose control standards for PLWH to prevent CVD?					
	11. How frequently should blood pressure and blood glucose be monitored in PLWH?					
B Drug Management	12. Which ART regimens may interact with statins?					
	13. What are the key considerations when co-administering rilpivirine with other cardiovascular medications such as calcium channel blockers?					
	14. What are the risks of drug interactions between tenofovir and diuretics?					
	15. How do protease inhibitors (PIs) in ART regimens influence the effectiveness of oral hypoglycemic agents such as sulfonylureas and metformin?					
	16. Which ART regimens should be avoided in HIV patients at high risk ofCVD?					
	17. Which ART options may contribute to increased LDL-C levels?					
	18. Which ART regimens are most likely to cause weight gain?					
C Clinical Decision-Making	19. What are the preferred hypoglycemic treatments for PLWH who are at high					

First-Level Indicator	Second-Level Indicator	Importance (1-5)	Feasibility (1-5)	Answer Definiteness (1-5)	Clarity of Expression (1-5)	Revision Suggestions
	cardiovascular risk and have type 2 diabetes?<br>20. What are the stepwise management strategies for HIV-positive patients with varying blood pressure levels?<br>21. What are the indications for initiating lipid-lowering therapy in PLWH?<br>22. What lipid-lowering strategies are recommended for patients with different levels of renal dysfunction?<br>23. What are the causes and management strategies for central adiposity in postmenopausal women receiving dolutegravir-based ART?					
D Case Analysis	24. A 45-year-old male with a 10-year history of HIV infection, on ART (EFV+TDF+FTC-based regimen). Current immune status: CD4+ 580 cells/μL, HIV RNA persistently <20 copies/mL (for 3 years). Persistent dyslipidemia (uncontrolled for 3 years): TC 5.2 mmol/L, LDL-C 3.4 mmol/L, HDL-C 1.0 mmol/L, TG 1.8 mmol/L; clinic BP 130/85 mmHg, home-measured average BP 128/82 mmHg; FPG 5.8 mmol/L, HbA1c 5.6%; BMI 26 kg/m², waist circumference 94 cm, eGFR 92 mL/min/1.73m². Framingham 10-year risk score 12%, coronary artery calcium (CAC) score 50. History of smoking (10 years, 20 pack-years), quit 2 years ago; sedentary office job, fast-food-based diet.<br><br>(1) What are the contributors to dyslipidemia in this patient? Is ART implicated?<br>(2) Does this patient meet indications for initiating lipid-lowering therapy?<br>(3) Design a lipid-lowering regimen including drug selection, dosing, and monitoring plan.<br>(4) Propose non-pharmacologic strategies to improve cardiovascular risk in this patient.					

First-Level Indicator	Second-Level Indicator	Importance (1-5)	Feasibility (1-5)	Answer Definiteness (1-5)	Clarity of Expression (1-5)	Revision Suggestions
	25. A 58-year-old woman with a 15-year history of HIV infection. Initial ART regimen: LPV/r + TDF/FTC (continued for 13 years). Switched to DTG + 3TC 2 years ago due to mixed hyperlipidemia (peak values: TC 7.2 mmol/L, LDL-C 5.0 mmol/L, TG 4.8 mmol/L). Concurrent conditions: Type 2 diabetes (5-year duration, HbA1c 7.5%), currently on metformin 1000mg bid; hypertension (8-year duration, BP 140-150/85-95 mmHg), on amlodipine. Latest fasting lipids: TC 6.0 mmol/L, LDL-C 4.0 mmol/L, HDL-C 1.1 mmol/L, TG 2.5 mmol/L; renal function: eGFR 68 mL/min/1.73m², UACR 32 mg/g. Cardiovascular risk scores: Framingham 28%, ASCVD 10-year risk 20%, coronary artery calcium (CAC) score 450. BMI 28 kg/m², sedentary lifestyle, waist circumference 92 cm.<br><br>(1) Based on CVD risk stratification tools (e.g., Framingham, ASCVD, CAC), how should this patient's risk be categorized?<br>(2) What is the target LDL-C for this patient?<br>(3) If statin monotherapy fails to achieve target, what are appropriate combination therapy strategies?<br>(4) Assess the justification for the simplified DTG+3TC regimen considering viral suppression, metabolic effects, and drug interactions.					

Table S4 Reference Answer Evaluation Form

Questions	Reference Answer Reasonableness (1-5)	Guideline Concordance (1-5)	Suggestions
Q1			
Q2			
Q3			
Q4			
Q5			
Q6			
Q7			
Q8			
Q9			
Q10			
Q11			
Q12			
Q13			
Q14			
Q15			
Q16			
Q17			
Q18			
Q19			
Q20			
Q21			
Q22			
Q23			
Q24			
Q25			

The reference answers for the corresponding questions are provided in the Appendix.

End of Form — Thank You for Your Support!
