# Supplementary material for: Comparative Performance of AI Models and Clinicians in Evidence-Based Cardiovascular Disease Management for People Living With HIV: Comparative Study
Source: J Med Internet Res. 2026 Aug 10;28:e89858. doi: 10.2196/89858 (PMC13456308; doi:10.2196/89858)
Supplement: Supplementary file 3 [file jmir-v28-e89858-s003.pdf]

Hello. This interview only covers CVD risk assessment and management for people living with HIV. All responses should be delivered verbally during our conversation. You are prohibited from accessing any external resources throughout the entire interview, including mobile phones, computers, printed guidelines, academic papers, and other reference materials; you may only answer based on your professional memory.

You may refer to the four authoritative clinical guidelines when responding. If conflicting recommendations exist across documents, you must follow this strict priority hierarchy:

- Chinese Guideline for the Primary Prevention of Cardiovascular Diseases
- Chinese Guidelines for the Diagnosis and Treatment of HIV/AIDS
- European 2023 HIV Cardiovascular Guidelines
- US 2024 HIV Cardiovascular Management Guidelines

You may voluntarily specify which guideline your opinions rely on in your answers.

All questions are divided into four fixed categories with an unchanging order:

- Basic knowledge (CVD risk assessment, behavioral lifestyle, health indicator control)
- Medication management (drug-drug interactions, adverse drug reactions)
- Clinical decision-making
- Case analysis

I will read each question aloud one at a time; I will only move to the next question after you fully finish your response. The total interview duration should be controlled within 60 minutes. Do not look up any reference materials mid-interview. The whole conversation will be audio-recorded for research analysis purposes. Do you fully understand all rules and agree to participate in this interview?
